# Supplementary material for: Geneticin reduces mRNA stability
Source: PLoS One. 2022 Jul 28;17(7):e0272058. doi: 10.1371/journal.pone.0272058 (PMC9333311; doi:10.1371/journal.pone.0272058)
Supplement: Attachment — Submitted filename: ResponseToReviewers_June.docx [file pone.0272058.s006.docx]

*We greatly appreciate the feedback from Reviewers 1-3 on this second consideration of our manuscript. We have attempted to address each reviewer’s comments in the indicated sections of our manuscript (see below, italicized). Our manuscript has been greatly improved by your careful reading and critical feedback. We greatly appreciate the thoughtfulness of your comments.*

**From the Editor:**

All changes requested by reviewers 1 and 2 can be made by changing the text to acknowledge caveats or add explanations as suggested. No new experiments will be necessary. Once the revised manuscript is submitted, an additional round of peer review may not be needed and the decision can be made at the editorial level.

*As indicated above, we have modified the manuscript in an attempt to address the remaining concerns of each reviewer. We have indicated the changes made below.*

**From Reviewer 1:**

Durmaz et al has satisfactorily address all the queries raised by this reviewer either by providing the justification to our comments or by mentioning the caveats and limitations of the experimental approach within the text. Therefore, considering the scope of the focused manuscript this reviewer recommends publication in the journal.
However, this reviewer has some further questions that requires justifications.

Major comments:
1. Line 158, figure number 1C-D, the polyribosome sedimentation was carried out after inhibiting the translation reaction at 15 minutes to trap the mRNAs in the initial phase of firefly luciferase production. This reviewer has no objection about the time points taken. Reviewer is just curious that the difference of mRNA degradation in G418 vs untreated (Figure 1B) is very prominent at t=20 and 30, then why the authors decided to go with 15 minutes of translation reaction followed by polyribosome sedimentation. The authors mention in the line 108-110, that “in vitro, we observe that G418 likely acts independently of ribosome collisions.” What if the timepoints of t=20 or 30 minutes could have roughly provided us the connection between G418 and ribosome collision. Without which the above statement remains void and should be re-written.

*In performing the experiment this way, our chief objective was to interrogate ribosome-bound mRNAs prior to significant differences in mRNA levels. We selected the 15 min time-point since that time allowed ribosome loading onto mRNAs, but was likely concurrent with their initial decay. As the reviewer notes, between 10 and 20 min (and before to some degree), we observe reduction in mRNA levels implying that mRNAs are decaying within that time window. We have updated the text to make this point more explicitly (see lines 158 - 59).*

2. The sedimentation assay (Figure 1D and 1E) was carried out to check the relative abundance of luciferase mRNA throughout the polysome fraction. From the result it was evident that the mRNA is present mostly in the monoribosomal fraction. But the authors did not mention the proper method to quantify the mRNAs. Did the author took ribosomal rRNA to measure relative abundance of luciferase mRNA? Or is it relative to each fraction of the polysome profile? It would be very informative if authors can describe this information in the method section.

*As indicated in the revised Methods, normalization was accomplished using a spike-in mRNA since various polysome fractions have different levels of rRNA. The Methods are changed in line 363 to indicate this point.*

Minor comments:

*We have made the changes in the text where indicated below.*
1. Incorporate the G418 (Geneticin) in the introduction as the title suggest and it would be informative to a novice reader. *(lines 103 - 107)*
2. Line 233-234: repetitive statement. *Removed.*
3. Interspersed grammatical errors. A thorough reading of the manuscript should take care of it.

*Edited throughout.*

**From Reviewer 2:**

- Introduction: It is important to clearly state in which organism the findings described in the introduction were obtained as there can be mechanistic differences between yeast, insects and mammals. As an example, the work describing the role of the Ccr4-Not complex at ribosomes with unoccupied A sites was performed in yeast.

*This is an excellent point. We have attempted to specify which systems were used to make the observations in the Introduction. Changes are indicated throughout using track changes.*

- Line 149-153 (Figure S1) : The authors tested whether G418 could delay elongation in RRL by monitoring luciferase expression levels (upon adding high concentrations of G418 and other translation inhibitors) using western-blotting. However, G418 induces amino-acid misincorporation and thus (at high concentrations), could possibly change the epitope recognized by the luciferase antibody. A better proxy to monitor this would be to perform in vitro translation experiments in the presence of S35-Methionine in order to quantify luciferase expression more accurately.

*Absolutely, labeling with ^35^S-Met would be ideal. However, we do not currently have a phosphorimager or access to a dark room for X-ray film development. That necessitates the use of antibodies or firefly luciferase activity measurements (which were used and are now indicated the in Fig. S1 legend). That being said, the activity that we do observe obeys delayed kinetics. The point is certainly valid, however, so we have modified the text in lines 149 - 51 to discuss this caveat and to be more explicit about how firefly luciferase protein levels were monitored.*

**From Reviewer 3:**

I commend the authors for submitting a greatly improved manuscript. They have de-emphasized and/or removed the unsupported text and have satisfactorily addressed this reviewer's core concerns.

While the authors have not definitively identified the mechanism by which G418 reduces the stability of the reporter RNAs (which is beyond the scope of this manuscript -as far as this reviewer is concerned-) they have effectively ruled out certain possible mechanisms.

On that note, I commend the authors for their use of polysome gradients to address my suggestion of ribosome collisions as the likely mechanism. Their data show nearly a complete absence of polysomes and a strong monosome peak. Simply, ribosomes can't collide when each mRNA is only translated by a single ribosome. It's a clever way to test my suggestion. As they show their polysome data do capture the regions of the gradient that span from 40S peak through part of the polysome region are convincing enough for this reviewer, they aren't perfect. (see below)

My lab regularly runs polysome gradients (using the same sucrose concentrations, and rotor) as part of our work. Simply, by this reviewer's experience their centrifugation conditions (10-50% gradients, centrifuged @ 39,000 RPM for 3 hours in an SW-41 rotor) likely resulted in the heaviest ribosomes pelleting. My lab uses very similar conditions, but we only spin samples for 2 hours (not 3). Granted, my lab's polysome results are all based on cell lysates as opposed to an in vitro translation system & there are differences between systems, but I'm fairly certain that the author's data only includes the polysome region corresponding to the position where polysomes corresponding to maybe the 5 or 6 ribosomes would be. The very long distance between the RNP peak at the start of the gradient and the monosome peak speaks to this as well.

Despite this concern, as I said above, I find these data (especially the absence of any di-ribosome peak -which, had it been there, would have been captured in their experiment) convincing enough to support publication of this manuscript.

*Although not specifically requested, we do now point out that our polysome profiles may exclude very large complexes of ribosomes and mRNA given how we prepared the samples. Those changes are made in lines 164 - 6.*

On a final note, I also encourage the authors to submit higher quality figures for publication. They are quite pixellated on my display and when I printed them out on hardcopy.

*We have attempted to upload higher resolution images.*
